# A dosimetry technique for measuring kilovoltage cone‐beam CT dose on a linear accelerator using radiotherapy equipment

**DOI:** 10.1120/jacmp.v15i4.4658

**Published:** 2014-07-08

**Authors:** Daniel Scandurra, Catherine E. Lawford

**Affiliations:** ^1^ Radiation Oncology Department Olivia Newton‐John Cancer and Wellness Centre, Austin Health Heidelberg Australia; ^2^ Sunshine Hospital Radiation Therapy Centre St Albans Australia

**Keywords:** CBCT, dose length product, dose length integral, DLP

## Abstract

This work develops a technique for kilovoltage cone‐beam CT (CBCT) dosimetry that incorporates both point dose and integral dose in the form of dose length product, and uses readily available radiotherapy equipment. The dose from imaging protocols for a range of imaging parameters and treatment sites was evaluated. Conventional CT dosimetry using 100 mm long pencil chambers has been shown to be inadequate for the large fields in CBCT and has been replaced in this work by a combination of point dose and integral dose. Absolute dose measurements were made with a small volume ion chamber at the central slice of a radiotherapy phantom. Beam profiles were measured using a linear diode array large enough to capture the entire imaging field. These profiles were normalized to absolute dose to form dose line integrals, which were then weighted with radial depth to form the DLP_CBCT_. This metric is analogous to the standard dose length product (DLP), but derived differently to suit the unique properties of CBCT. Imaging protocols for head and neck, chest, and prostate sites delivered absolute doses of 0.9, 2.2, and 2.9 cGy to the center of the phantom, and ${\text{DLP}}_{\text{CBCT}}$ of 28.2, 665.1, and 565.3 mGy.cm, respectively. Results are displayed as dose per 100 mAs and as a function of key imaging parameters such as kVp, mAs, and collimator selection in a summary table. ${\text{DLP}}_{\text{CBCT}}$ was found to correlate closely with the dimension of the imaging region and provided a good indication of integral dose. It is important to assess integral dose when determining radiation doses to patients using CBCT. By incorporating measured beam profiles and DLP, this technique provides a CBCT dosimetry in radiotherapy phantoms and allows the prediction of imaging dose for new CBCT protocols.

PACS number: 87.57.uq

## I. INTRODUCTION

In line with recent concerns regarding the amount and frequency of radiation doses administered from radiological examinations, in particular the prolific use of computed tomography (CT), the medical community is discussing guidelines and protocols for the safe and effective use of ionizing radiation for cone‐beam computed tomography (CBCT) in radiation therapy. This poses a challenge unique to radiotherapy, as the biological consequences of adding imaging doses to a patient already receiving tumoricidal levels of radiation are not well understood. A major focus for the optimization of patient dose in diagnostic procedures is reducing the risk of cancer induction, yet this assessment is particularly difficult in radiotherapy where cancer is already present.^(1)^ Indeed, there are many questions surrounding CBCT dosimetry without satisfactory answers, and this has contributed to a lack of consensus among the radiotherapy community regarding appropriate measurement techniques.

Central to the discussion on effective use of imaging is the relationship between dose and image quality. It is inherently implied that imaging improves treatment outcome and, therefore, dose from imaging is a necessary cost; however, the amount of dose delivered to achieve the desired outcome must be optimized.^1^, ^2^ In order to evaluate this cost‐benefit relationship adequately, the ‘cost’ must be recorded accurately and reported consistently. It is prudent to establish a robust procedure for recording and reporting of imaging dose, and the AAPM TG‐111 report has recently established such a standard process for diagnostic CT.^(3)^ These concepts are not, however, directly translatable to radiotherapy CBCT.

Consequently, published literature shows much variation in the measurement and reporting of radiotherapy CBCT doses, which makes it difficult to provide clear guidelines for the clinic.^4^, ^5^, ^6^, ^7^, ^8^, ^9^, ^10^, ^11^, ^12^, ^13^, ^14^ For instance, there are known problems with using conventional diagnostic CT dosimetry in the CBCT equipment used on linear accelerators in radiotherapy, due to important differences in beam geometry.^15^, ^16^ The Computed Tomography Dose Index with a 100 mm pencil chamber (CTDI_100_) was originally designed to predict the equilibrium dose at the center of a series of axial scans.^(17)^ This was done by measuring the integral of the single slice dose from a narrow axial scan using a chamber long enough to capture the entire dose, including scatter tails. Initial approaches to CBCT dosimetry were an extension of this concept, but it has since been shown to be inadequate. Two main tenets of CTDI dosimetry are no longer valid: a 100 mm pencil chamber does not capture the entire dose from one scan and, therefore, does not give an accurate indication of dose at the center of the profile, and the CBCT profile shape cannot be approximated by the superposition of a series of axial slice profiles.

More robust techniques have since been developed using Monte Carlo simulation on mathematical phantoms or on actual patient data, as well as comprehensive point dose analysis using embedded dosimeters in anthropomorphic phantoms. These two techniques, in particular, have allowed for the reporting of integral dose, as well as individual organ doses, leading to a calculation of whole body effective dose. Not all clinics, however, have the resources necessary to perform Monte Carlo modeling or access to anthropomorphic phantoms to enable characterization of their own CBCT systems and imaging protocols in this way.

The AAPM TG111 report has recommended that integral dose be included in the investigation of imaging dose and imaging protocols should be optimized to avoid unnecessary exposure to tissues further from the target. Point doses do not vary significantly with scan length and so are not sufficient to assess and compare the integral dose of different protocols. In linear accelerator CBCT, integral dose is highly dependent upon the length of collimation along the patient.

The primary objectives of this report were to address several challenges: how to characterize and report on radiotherapy CBCT imaging doses whilst adhering to recommendations from TG‐111, to perform these measurements for our own department scan protocols, and to do this using our existing radiotherapy dosimetry equipment.

In this paper, the methods incorporate elements of the CTDI dosimetry concept, but with variations which address the unique properties of linear accelerator‐based CBCT, with particular regard to the two issues mentioned already: accurately measuring central plane dose and the determination of the dose profile shape. The developed technique also provides sufficient data to predict dose for new imaging protocols.

1. Absolute dose measurements are made at central and periphery positions in two CIRS phantoms (”head and neck”, and “body”) using CBCT acquisitions, according to TG61 recommendations.^(18)^
2. Beam profile measurements are made using a linear diode array at depths matching the absolute dose measurements made in the CIRS phantoms.
3. The beam profile is normalized to the absolute dose measured at the corresponding position in the CIRS phantom by correcting for the ratio of the absolute dose to the dose at the center of the beam profile. This absolute dose profile is integrated to calculate DLI.
4. ${\text{DLP}}_{\text{CBCT}}$ is determined by taking a weighted average of central and peripheral DLIs. Integral dose can then be assessed in much the same fashion as DLP is used in conventional CT dosimetry, but derived from measurements tailored specifically for CBCT.

## II. MATERIALS AND METHODS

The measurements were performed on an Elekta Synergy linear accelerator (Elekta, Stockholm, Sweden) with a CBCT system that consists of a kV X‐ray source, a large area flat panel detector, and the X‐Ray Volumetric Imaging (XVI) software. Only a brief overview of the system is presented, as detailed descriptions have been discussed elsewhere.^12^, ^13^, ^19^ The kV imaging system is mounted perpendicular to the MV treatment axis, and is calibrated such that the imaging (kV) and treatment (MV) isocenters coincide.

The CBCT collimators are user selectable, depending upon the desired field of view (FOV). The small (S) collimators project a symmetrical field with a geometric FOV of 276.7 mm width at the isocenter. With the medium (M) or large (L) collimator, an asymmetric beam is projected to the offset panel position and can extend the FOV to 426.4 mm and 524 mm, respectively. Additionally, the longitudinal extent is selectable to field lengths of 35.2 mm, 135.4 mm, 178.7 mm, and 276.7 mm through a choice of 2, 10, 15, and 20 collimators. A collimator is, therefore, known by these two dimensions and will be referred to as S10, M10, M20, and so forth. Table 1 displays the XVI factory protocols and scan parameters that were used for these investigations.

**Table 1:** Characteristics of factory XVI volume view protocols and scan parameters. All protocols use clockwise gantry rotation with no filter (F0)

	*H&N*	*Chest*	*Prostate*
Tube voltage (kVp)	100	120	120
Tube current (mA) per frame	10	25	40
Exposure (ms) per frame	10	40	40
Frames	361	650	650
Total mAs	36.1	650	1040
Additional filter	none	none	none
Collimator	S20	M20	M10
CBCT start angle (°)	70	270	270
CBCT stop angle (°)	270	270	270
Total acq. Angle (°)	200	360	360

### A. Point doses

#### A.1 Absolute dosimetry – TG‐61 methodology

**Figure 1:**
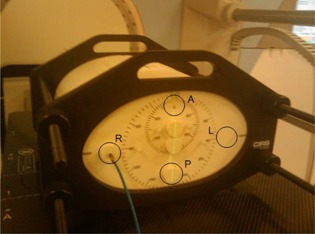
Absolute dose for the 120 kV CBCT protocols involved the use of a CIRS body phantom. Doses were recorded at central and peripheral (A, P, R, L) locations.

Absolute doses were recorded at a range of collimator and beam energy combinations at the center and periphery of both head and body phantoms. The ‘head phantom’ (CIRS 002HN, Norfolk, VA) is a cylindrical phantom 16 cm in diameter and 30 cm long, Measurements were made at the center and at 2 cm depth from the anterior (A), posterior (P), left (L), and right (R) of the phantom (position descriptors using a supine, head‐first patient setup). The ‘body phantom’ (CIRS 002H9K), shown in Fig. 1, is 20 cm high, 30 cm wide, and also 30 cm long, with measurements made at the center, and at 5 cm depth from the top, bottom, and sides. Both phantoms have a physical density of $1.039\;g/{\text{cm}}^{3}$ and relative electron density of 1.001.

To simulate head and neck CBCT, the head phantom was centered at the isocenter and scanned using the S10 and S20 collimators and a 100 kVp beam. Half‐rotation $\left(∼200^{\circ }\right)$ scans are commonly used for head and neck CBCT and were therefore used here, as well as a full rotation scan.

For the body phantom measurements, the tube voltage was set to 120 kVp. Absolute dose was recorded for a range of available collimators: S10, S20, M10, M15, M20, L10, and L20 using full 360° rotation.

For each exposure the XVI system was set to deliver 1000 mAs for adequate signal to noise; however, the displayed results are subsequently scaled back to give dose per 100 mAs. Dose linearity was checked using the same setup for a clinical range of mAs. Absorbed dose measurements were made according to TG‐61 recommendations^(18)^ with an NE2571 thimble chamber (Thermo Electron Corporation, Runcorn, Chesire, UK) calibrated at a secondary standard dosimetry laboratory over a range of diagnostic energies and exhibiting an energy dependence of less than 2% at the beam qualities of interest. The TG‐61 protocol gives the following formula for determining the absorbed dose to water for 100‐300 kV tube potentials:

(1) $$D_{w}=MN_{k}P_{Q,cham}{\left[{\left(\frac{{\bar{\mu }}_{en}}{\rho }\right)}_{air}^{water}\right]}_{water}$$

where *M* is the electrometer reading corrected for ion recombination, polarity, temperature, pressure, and electrometer response; $N_{k}$ is the air kerma calibration factor for the user beam quality Q; $P_{Q,cham}$ is the overall chamber correction factor accounting for change in the chamber response due to changes in energy and stem effects; and ${{\left[({\bar{\mu }}_{en}/\rho \right)}_{air}^{w}]}_{w}$ is the water‐to‐air ratio of the mean mass energy‐absorption coefficients, measured in water. Beam quality was determined by measuring HVL, as described in TG‐61. In addition, corrections were made to compensate for differences in field size and measurement depth from the TG‐61 reference conditions, where needed.^(20)^

### B. Dose line integral (DLI) and ${\text{DLP}}_{\text{CBCT}}$ calculation

#### B.1 DLI

The dose to the volume of a phantom cannot be adequately described by a single point‐dose measurement at the center. To enable the assessment of integral dose within the entire imaging volume, dose information along all off‐axis points is required and the area under the curve determined. Ideally, this dose profile would be acquired with an ion chamber positioned in the phantom and incrementally translated along the length of the scan, with a full CBCT rotation at each point. These profiles should be acquired at both central and peripheral locations. However, a very high mAs is required to achieve a reasonable signal‐to‐noise ratio, particularly at the center of the phantoms, and the subsequent heat generation is problematic for the air‐cooled kV tube used in the XVI system. Combined with the number of measurements needed to provide a reasonable approximation of the profile shapes, this approach is impractical for repeated profile measurements. As an alternative, a linear diode array was validated and used to instantaneously acquire the full profile shape.

In order to validate the linear diode array, dose profiles were acquired using a small volume ion chamber (CC13; IBA, Germany) along the center and periphery of both phantoms by incrementally moving the phantom through the scan region. A full CBCT rotation was delivered at each point using the M15 collimator, 100 kV for the head phantom and 120 kV for the body phantom to reflect clinical practice. The periphery locations are 2 cm depth for the head and neck phantom, and 5 cm depth for the body phantom. For each pair of profiles (center and periphery), the scans were normalized to the central axis and compared. It was seen that the area under each profile differed by less than 5% for both phantoms and, while the profile shape itself showed slight variations, each pair of chamber measurements along the phantom matched to within ±5%.

The similarity of dose profiles at the center and along the periphery was surprising and is likely due to the average depth of the peripheral chamber location over a complete rotation being close to the depth of the center. This result was used to simplify data acquisition by subsequently only measuring profiles at the depth of the center of each phantom.

**Figure 2:**
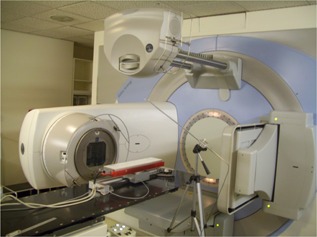
The BMS 96 linear diode array positioned at 100 cm source‐to‐detector distance along the longitudinal axis of the X‐ray beam and location of reference diode. PMMA buildup and solid water were used to customize measurement depths.

These profiles were then compared to those obtained using a BMS 96 linear diode array (Schuster Medizinische Systeme, Forchheim, Germany) with a vertically incident (i.e., stationary) irradiation (Fig. 2). The array contains 88 diodes, each with an active area of 2.5 mm^2^ and spaced 5 mm apart along a length of 440 mm. It has an inherent buildup of 1 cm and profiles can be taken at any depth with the addition of solid water buildup.

The array is mounted on a motor drive mechanism which allows it to be shifted longitudinally. When centered along the beam central axis, the array is of sufficient length to capture the entire CBCT field at most depths. For the combination of large length scans at greater depths, the centered array was not completely able to measure the full extent of the scatter tail. For these scans, the array was shifted in each direction to capture the full tail, and the resultant profile is a combination of three scans superimposed together.

An array calibration was performed for each measurement profile. The calibration procedure shifts the array through the full length of the beam, exposing each individual detector to the same portion of the beam. The calibration was performed with the appropriate XVI collimator, kV energy, and depth of buildup subsequently used for the profile acquisition.

**Figure 3:**
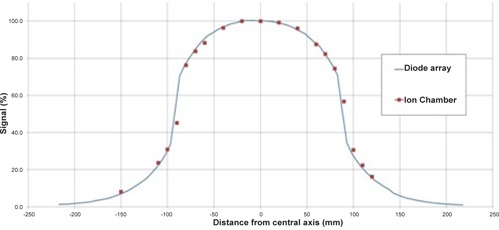
The profile generated by the linear diode array with 8 cm buildup compared to ion chamber (CC13) measurements at the center of the HN phantom for a CBCT acquisition with M15 collimator at 100 kV.

**Figure 4:**
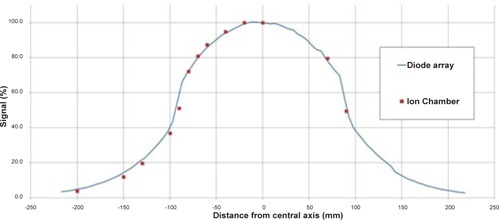
The profile generated by the linear diode array with 15 cm buildup compared to ion chamber (CC13) measurements at the center of the body phantom for a CBCT acquisition with M15 collimator at 120 kV

A reference diode was positioned within the beam to account for variations in tube output. The profiles were acquired at 8 cm (using 100 kV) and 15 cm deep (using 120 kV), by the addition of solid water, to coincide with the depth at the center of each phantom. As the gantry rotates, the center of the body phantom is not at a uniform depth from the surface; the maximum depth (15 cm) was chosen as the extra scatter gives a larger dose profile integral, giving a worst‐case integral dose. When normalized to the central axis, doses recorded at any position along the profile using the linear diode array differed by less than ±3% at 8 cm deep and by less than ±5% at 15 cm deep, compared to the ion chamber profiles, leading to a difference of less than ±5% in total profile area. These differences can be seen in Figs. 3 and 4 for scans taken with the M15 collimator and at 100 and 120 kV, respectively.

Having validated the use of a linear diode array for profile measurement using the M15 collimator, profiles were acquired for all other collimators at each depth (and corresponding kV). Each profile was then converted to an absolute dose profile by correcting for the ratio of the corresponding absolute dose, measured in the Materials and Methods section A.1 above, to the dose at the center of the beam profile. These profiles were then integrated to form the dose line integrals (DLI), with units of mGy.cm, indicating the total dose along the longitudinal line of equal depth within the phantom.

#### B.2 *DLP*_*CBCT*_ *calculation*

It is common practice to assume a nonconstant radial dose between the periphery and center of the phantom. CTDI protocols recommend weighting the peripheral and central doses by 2/3 and 1/3, respectively, which has been reported as a more accurate estimate than the equal weighting approach stated in TG‐111.^(21)^ This relationship is used in this work for both head and body phantoms, although the suitability of this approximation to an oval phantom is unproven.

The radially‐weighted DLI gives rise to the ${\text{DLP}}_{\text{CBCT}}$ (mGy.cm):

(2) $$DLP_{CBCT}=\frac{1}{3}DLI_{Centre}+\frac{2}{3}DLI_{Periphery}$$

The purpose of the ${\text{DLP}}_{\text{CBCT}}$ is to provide an indication of the average DLI in the phantom. It is analogous to the standard DLP that is familiar in diagnostic CT, but the CBCT subscript has been added to differentiate the method with which it has been derived. The standard DLP is based upon conventional CT dosimetry with 100 mm ion chambers and, as stated previously, this has been shown to be inadequate in this context. The ${\text{DLP}}_{\text{CBCT}}$, therefore, has the same units and properties as DLP, except the technique used is more appropriate for CBCT.

The ${\text{DLP}}_{\text{CBCT}}$ is a useful tool which allows for comparisons of CBCT protocols with each other and to other imaging modalities. Just as the absolute dose parameters are reported per 100 mAs, the ${\text{DLP}}_{\text{CBCT}}$ is also reported per 100 mAs.

## III. RESULTS

### A. Point doses

#### A.1 Absolute dosimetry – TG‐61 methodology

A first HVL of 5.75 mm and 6.80 mm Al was measured for the 100 kVp and 120 kVp beams, respectively, in close agreement with previously reported values.^5^, ^7^, ^12^

Linearity of the system was investigated and dose output as a function of tube current and exposure follows a highly linear relationship $\left(R^{2}>0.999\right)$.

The absorbed‐dose measurements in the body phantom are presented in Table 2 in cGy per 100 mAs. The doses distributed around the periphery of the phantom are relatively constant as expected, with a maximum at location R coinciding with the X‐ray tube position at the start and stop angles of the acquisition. The ratio of dose from the center to the periphery is higher than expected with conventional CTDI type phantoms. This is due to the 30 cm×20 cm oval shape of the CIRS body phantom, as compared with the standard 32 cm diameter CTDI cylinder. An increase in central dose of 10%–20% is observed between the 10 and 20 collimators for each FOV, as extra scatter is generated from increased field length. Posterior peripheral doses are lower due to attenuation through the couch top.

The absorbed dose in the head phantom was measured using the S10 and S20 collimators at 100 kVp for a full rotation and half‐rotation scan (as described in Table 1). The doses in the head phantom, presented in Table 3, are lower than those recorded in the body phantom due to the reduction of tube voltage to 100 kVp, despite the phantom being considerably smaller.

Interestingly, the central and average peripheral doses are similar for both the full and half‐rotation scans, just distributed differently. This is because the results are displayed per 100 mAs, and the total mAs is proportional to the total scan angle (i.e., approximately half the mAs is used for a half‐rotation scan). Total point doses would be much higher for the full rotation than for the half rotation. The average peripheral dose is used in the subsequent calculation of ${\text{DLI}}_{\text{periphery}}$, and it is worth noting that this figure may be significantly higher or lower at various points around the circumference during a half‐rotation CBCT.

**Table 2:** Absolute dose measurements in cGy per 100 mAs within the body phantom as a function of collimator and chamber position at 120 kVp tube potential. Peripheral chamber cavities are located 5 cm from the surface

	*Body Phantom, 120 kVp, cGy per 100 mAs, full rotation*						
*Collimator*	*S10*	*S20*	*M10*	*M15*	*M20*	*L10*	*L20*
Center	0.30	0.36	0.28	0.31	0.34	0.23	0.26
A	0.38	0.44	0.34	0.37	0.39	0.25	0.28
L	0.43	0.49	0.33	0.35	0.37	0.25	0.28
P	0.35	0.41	0.32	0.35	0.38	0.23	0.26
R	0.45	0.50	0.36	0.38	0.41	0.28	0.32
Avg. Periphery	0.40	0.46	0.34	0.36	0.39	0.25	0.28

**Table 3:** Absolute dose in cGy per 100 mAs within the CIRS head phantom as a function of collimator and phantom position at 100 kVp. Half–rotation is a 200° scan from $-20^{\circ }$ to 180°

*Head Phantom, 100 kVp, cGy per 100mAs*				
	*Full Rotation*		*Half Rotation*	
*Collimator*	*S10*	*S20*	*S10*	*S20*
Center	0.23	0.25	0.23	0.26
A	0.29	0.31	0.15	0.16
L	0.28	0.31	0.31	0.33
P	0.28	0.30	0.40	0.44
R	0.30	0.31	0.28	0.30
Avg. Periphery	0.29	0.31	0.28	0.31

### B. DLI and ${\text{DLP}}_{\text{CBCT}}$ calculation

Measured longitudinal profiles did not vary significantly with FOV; that is, S10, M10, and L10 profiles are interchangeable and so on for the 15 and 20 groups.

**Figure 5:**
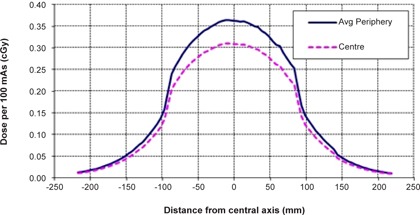
DLIs of CBCT scan using M15 collimator, 120 kVp in CIRS body phantom. Profiles are normalized to absolute dose within the body phantom measured at the center (5 cm) and around the periphery (15 cm depth) and are given per 100 mAs.

Renormalizing the profiles to the absolute dose from the Material and Methods section A.1 allows the DLI to be calculated. Knowing the CBCT preset and imaging region, the correct combination of dose and profile can be made. For example, take a CBCT preset for the pelvis using the M15 collimator, which corresponds to the use of 120 kVp and body phantom measurements. Table 2 shows the M15 body phantom scan delivers 0.31 cGy and 0.36 cGy, per 100 mAs, at the center and periphery locations, respectively. These point doses correspond to the 15 cm deep body phantom beam profile from Fig. 4 and these renormalized profiles are shown together in Fig. 5. Integration of the area under these profiles gives DLIs of 62.5 and 73.4 mGy.cm, respectively, and a ${\text{DLP}}_{\text{CBCT}}$ of 69.7 mGy.cm. Table 4 displays the ${\text{DLP}}_{\text{CBCT}}$ for each collimator and phantom combination, per 100 mAs.

**Table 4:** The final table of dose parameters listed per 100 mAs for each combination of collimator and phantom

*Summary: Doses per 100 mAs*						
		Point Dose (cGy)		*DLI (mGy.cm)*		
	*Collimator*	*Center*	*Avg. Periphery*	*Center*	*Avg. Periphery*	*DLP*_*CBCT*_ *(mGy.cm)*
Head & Neck Phantom 100 kV	S10	0.23	0.29	32.9	41.7	38.7
	S20	0.25	0.31	68.3	83.3	78.2
Body Phantom 120 kV	S10	0.30	0.40	51.8	69.0	63.2
	S20	0.36	0.46	100.8	127.1	118.2
	M10	0.28	0.34	48.1	57.6	54.4
	M15	0.31	0.36	62.5	73.4	69.7
	M20	0.34	0.39	93.4	107.0	102.3
	L10	0.23	0.25	38.6	42.7	41.3
	L20	0.26	0.28	72.5	78.3	76.3

### C. Summary of results

A summary of dose parameters are listed per 100 mAs in Table 4. Making use of the linearity of dose response, this table provides a quick way to determine CBCT dose for new imaging protocols. Dosimetry can be predicted by defining the region to be imaged, selecting the appropriate collimator, and then multiplying the listed parameters by the prescribed mAs.

### D. Calculation of dose for factory CBCT protocols

Table 5 lists the doses recorded for CBCT of three main treatment sites using factory XVI protocols with relevant scan parameters, as listed in Table 1.

**Table 5:** Summary of doses recorded for three Elekta XVI factory protocols

	*Head and neck*	*Chest*	*Prostate*
Phantom (kVp)	Head (100)	Body (120)	Body (120)
Point dose (cGy) (center)	0.09	2.19	2.94
Point dose (cGy) (Avg. periphery)	0.11	2.51	3.51
DLPCBCT (mGy.cm)	28.2	665.1	565.3

#### D.1 Head and Neck

Scaling back the S20 doses for the head phantom to 36.1 mAs gives point doses of 0.09 to 0.11 cGy and ${\text{DLP}}_{\text{CBCT}}$ of 28.2 mGy.cm. The half‐rotation scan introduces a complexity that is not accounted for by the final table. There will be regions around the periphery of the phantom that contain much higher or much lower doses than the average. This can be used for sparing organs at risk by customizing protocol scan angles for specific treatment sites. Current scan angles would reduce dose to the thyroid (a major contributor to effective dose in the head and neck region) and eyes, while for esophagus and larynx sites, it may be beneficial to scan across the anterior portion of the patient, reducing dose to the spinal cord.

#### D.2 Chest

The chest protocol uses the M20 collimator at 120 kVp with a total of 650 mAs over a full rotation. This results in point doses of 2.19 to 2.51 cGy, and ${\text{DLP}}_{\text{CBCT}}$ of 665.2 mGy.cm.

#### D.3 Prostate

Scaling the M10 results to 1040 mAs results in point doses of 2.94 to 3.51 cGy and a ${\text{DLP}}_{\text{CBCT}}$ of 565.3 mGy.cm. The prostate preset uses high mAs to provide adequate soft‐tissue information. This leads to the highest point doses of all factory protocols; however, the ${\text{DLP}}_{\text{CBCT}}$ is still less than the chest region due to its shorter scan length. It is clear that appropriate collimator selection plays a large role in minimizing the radiation burden from CBCT.

## IV. DISCUSSION

Taking into account the differences in experimental setup, such as mAs and phantom dimensions, the point doses reported in this work fall within the range of previously published values and closely correlate to those undertaken using Elekta XVI specifically.^4^, ^7^, ^12^, ^14^ The low dose recorded using the head and neck protocol is not only a consequence of the smaller patient cross section in the region; typical CBCT registration uses bony anatomy which requires far less exposure than other treatment sites where soft tissue delineation is crucial. The prostate is surrounded by soft tissue and has been shown to move independently of the pelvic bones; therefore, it requires better image quality and subsequently a substantial increase in dose for its imaging protocol. Therefore, any reduction of dose must be made within the constraints of clinically acceptable image quality, and the process of optimization is a fine balance between the two.

Point doses can be used to assess the risk of toxicity to individual tissues, whether the target or adjacent healthy structures. At particular risk are organs of a serial nature, where the addition of the imaging dose may increase the likelihood of exceeding the threshold for damage. In our center, daily CBCT imaging for a 78 Gy prostate patient would result in 39 fractions of extra radiation totaling ∼1.4 Gy at 2 cm below the skin and 1.1 Gy at the isocenter, delivering a substantial dose that is generally not accounted for by the clinician. It should also be noted that the reported point doses are absorbed dose to water, and dose to bone would be much higher due to an increase in photoelectric interactions for kilovoltage beams. Using Monte Carlo models, some publications have calculated a relative increase of ~ three to four times the dose to tissue in bone, a substantial amount to vulnerable tissues, such as the mandible.^22^, ^23^

However, the central axis point doses measured here give no indication of the integral dose deposited in the patient, which depends on scan length, as well. Longitudinal beam profiles show that dose within the imaging field can vary significantly from the central axis point dose, particularly at greater depths. Beam profiles are, therefore, necessary to provide accurate indication of integral dose.

The combination of beam profiles with point dose leads to the use of DLIs and the ${\text{DLP}}_{\text{CBCT}}$ technique. Similar in function but derived very differently to DLP in CT, the ${\text{DLP}}_{\text{CBCT}}$ provides a concise metric that incorporates several system parameters at once: length and shape of the CBCT beam profile due to collimator selection, the radial dose distribution in a phantom, and absolute dose as a function of tube voltage (kVp) and tube current (mAs). As an example of the necessity to evaluate integral dose, point doses at the center of the body phantom show an increase of only 20% when doubling the field length (e.g., from M10 to M20), yet the ${\text{DLP}}_{\text{CBCT}}$ increases approximately 200%. This therefore leads to a more thorough understanding of the dose to the whole patient than central axis point doses alone. Other uses of ${\text{DLP}}_{\text{CBCT}}$ (similar to the use of DLP in diagnostic CT) include characterizing CBCT systems, maintaining system constancy through ongoing quality assurance, and patient dose optimization through the comparison of different imaging parameters and protocols.

Further, integral dose (i.e., the total energy absorbed in the phantom) can be calculated from ${\text{DLP}}_{\text{CBCT}}$ by multiplying the phantom cross‐sectional area and tissue density.^(3)^ Integral dose is relevant to radiation risk and, therefore, may be beneficial for physicists and clinicians seeking to assess the future health impact of CBCT protocols. Effective dose, another quantity associated with radiation risk and more commonly used in radiation protection, can be derived from integral dose by investigating the dose to specific tissues irradiated in the imaging region. Effective dose should not be used to determine risk from individual procedures to individual patients, particularly in radiotherapy where some of these tissues will already be receiving tumoricidal levels of radiation by the treatment beams. However, effective dose may be a useful way of comparing the population‐wide potential health impact of scanning protocols from different imaging modalities.

The measured line profiles show that CBCT dose is not constant across the length of the image. Particularly at greater depths, even regions close to the center of the field are rounded (see Fig. 4). This is a clear example of how a 100 mm pencil ion chamber, as used in diagnostic CT, will have a large volume average effect and underestimate the true central dose. Additionally, the shape of the profile is such that the length of the field cannot be accurately described by the use of a nominal FOV or FWHM. While it is common to specify the length of a therapy field by the FWHM, it is clear that significant dose is deposited outside of this region in CBCT. If the nominal FOV or FWHM was used rather than the measured profile, the integral dose would not be accurate. It is therefore preferable to scan the entire length of the dose profile and normalize to absolute dose measured with a small volume ion chamber, as this work suggests.

Data from Table 5 enables the calculation of imaging dose for a new CBCT preset without needing to make specific measurements. However, use of a different kVp or filter (such as the bowtie filter) would require some new point dose and profile data.

## V. CONCLUSIONS

A range of measurements have been performed for CBCT on an Elekta XVI system. Point dose was measured using an NE2571 cylindrical chamber with TG‐61 methodology in two CIRS phantoms and reported per 100 mAs. Longitudinal beam profiles were acquired using a linear diode array and, after normalizing the profiles to absolute dose, calculations of DLI and ${\text{DLP}}_{\text{CBCT}}$ were made. The ${\text{DLP}}_{\text{CBCT}}$ can be used to assess each CBCT dose footprint within the phantom, incorporating the effects of collimation, phantom geometry, and tube output.

All the parameters have been presented in a summary table that allows quick dosimetry evaluations and comparisons for new and existing CBCT protocols. Absolute dose for Elekta XVI factory protocols ranged from 0.09 to 0.11 cGy, 2.19 to 2.51 cGy, and 2.94 to 3.51 cGy for head and neck, chest, and prostate protocols, respectively. ${\text{DLP}}_{\text{CBCT}}$ for each protocol was 28.2, 665.2, and 565.3 mGy.cm, respectively.

With the increasing frequency of CBCT as a preferred modality in image‐guided radiotherapy procedures, there is a growing clinical need to assess patient imaging dose. There is a known cost‐benefit relationship between image quality (and presumably treatment quality) and imaging dose, yet a thorough understanding of the ‘cost’ has been difficult to develop. One such reason is the lack of consensus for a suitable and universal dosimetry standard within the radiotherapy community, and so a simple technique has been developed in this paper that may be a further step towards meeting the clinical need for integral dose in CBCT.
